# Formulation and Bioavailability of Novel Mucoadhesive Buccal Films for Candesartan Cilexetil in Rats

**DOI:** 10.3390/membranes11090659

**Published:** 2021-08-26

**Authors:** Omar Y. Mady, Mahmoud M. A. Abulmeaty, Ahmed A. Donia, Abdulaziz A. Al-Khureif, Adam A. Al-Shoubki, Manal Abudawood, Doaa A. Abdel Moety

**Affiliations:** 1 Department of Pharmaceutical Technology, Faculty of Pharmacy, Tanta University, Tanta 31511, Egypt; 2 Department of Community Health Sciences, College of Applied Medical Sciences, King Saud University, Riyadh 11433, Saudi Arabia; 3 Department of Medical Physiology, School of Medicine, Zagazig University, Zagazig 44519, Egypt; daabdelmoeti@zu.edu.eg; 4 Department of Pharmaceutical Technology, Faculty of Pharmacy, Menofia University, Shebin El-Kom 13829, Egypt; ahmed.atef@phrm.menofia.edu.eg; 5 Dental Health Department, College of Applied Medical Sciences, King Saud University, Riyadh 10219, Saudi Arabia; aalkhuraif@ksu.edu.sa; 6 Department of Pharmaceutics, Faculty of Pharmacy, Omar Al-Mukhtar University, Al-Bayda 0463, Libya; Adim.alsanousi@omu.edu.ly; 7 Department of Clinical Laboratory Sciences, College of Applied Medical Sciences, King Saud University, Riyadh 11433, Saudi Arabia; mabudawood@ksu.edu.sa

**Keywords:** candesartan cilexetil, bioadhesive buccal film, pharmacokinetics

## Abstract

Candesartan cilexetil (CC) is an antihypertensive drug. It has low solubility and faces hepatic first-pass metabolism after oral ingestion. We formulated bioadhesive buccal films and studied the respective drug pharmacokinetics. Different bioadhesive films were prepared (40, 80, 120, 160, 200, and 240 mg CC per film) by using the solvent casting method. The drug concentrations used affect the drug entrapment mechanism, which was reflected in the film physicochemical properties like thickness, weight, drug content, bioadhesion, and drug release. Low drug concentration (F2, 40 mg per film) led to minute drug crystal dispersion while increasing the drug concentration (F7, 240 mg per film) showed drug crystal encapsulation, which affects the drug release. The drug pharmacokinetic from the prepared films was studied compared to the oral form by serial blood sampling via an inserted catheter in the carotid of rats. High-Performance Liquid Chromatography assay was used to measure the plasma concentration of CC in different forms. Compared to other films, the F2 showed the highest maximal concentration (Cmax) and the lowest elimination half-life (t_1/2_). Bioadhesion buccal film of CC has better bioavailability, especially at low concentrations. The ease, robustness, and ruggedness of the preparation suggests the same procedure for drugs like CC.

## 1. Introduction

Candesartan cilexetil (CC) is an antihypertensive drug and is also used for the management of many coronary heart diseases. It is a selective, reversible and competitive angiotensin II type-1 (AT1) receptor blocker [1]. The ester linkage of CC after oral administration will be hydrolyzed to form the active drug, candesartan [2,3]. The terminal elimination half-life of CC is about 5–10 h with total plasma clearance of 0.37 milliliter/min/ “ml/min/kg” and renal clearance of 0.19 mL/min/kg [4].

CC is, according to BCS, a class II drug. This class is a group of pharmaceutical active ingredients that is characterized by low solubility and high permeability. Since the drug should be soluble before absorption, this class has low bioavailability. The absolute bioavailability of CC is about 14–40% [1]. In addition, CC undergoes extensive first-pass metabolism in the liver which leads to also decrease in its bioavailability when administered as an oral convenient dosage form [5].

Nanotechnology is introduced as a promising solution for CC to solve its bioavailability problem (1). The drug delivery systems of nanoemulsions, dendrimers, niosomes, solid lipid nanoparticles (SLNs), polymeric nanoparticles, and nanostructured lipid carriers (NLCs) have been extensively investigated for the improvement of the bioavailability of antihypertensive drugs [6,7]. In addition, Ali et al. used different techniques like solid dispersions (using different polymers and solvent evaporation and fusion techniques), inclusion complexation with β-CD and HP-β-CD by co-evaporation technique, and nanoparticles of CC prepared by the solvent evaporation method using various polymers to enhance the bioavailability of candesartan cilexetil. The authors reported that the bioavailability of CC was significantly improved from ~15% to ~48% when formulated as solid dispersion with PVP K-90 with a 1:4 drug:polymer ratio [8].

Buccal drug delivery is a promising area for systemic drug delivery. The buccal drug delivery system is categorized, according to the site of drug action, into three categories which are sublingual delivery, buccal drug delivery, and local delivery. 1. Sublingual delivery in which the systemic delivery of drugs is through the mucosal membranes lining the floor of the mouth. 2. Buccal delivery, in which the drug administration through the mucosal membranes lining the cheeks (buccal mucosa). 3. Local delivery in which the drug is delivered into the oral cavity (local effect). Besides a buccal delivery system, a mucoadhesion buccal drug delivery system, which is the term used for materials that bind to the mucin layer of a biological membrane, is also developed. Buccal adhesive drug delivery systems include matrix tablets, films, layered systems, discs, microspheres, ointments, and hydrogel [9,10,11,12,13,14]. For local treatment of C. Albicans infection, Mady et al. [15] succeeded in preparing a buccal bioadhesive film which could be considered as a promising local treatment for oral candidiasis since oral candidiasis is a leading cause of morbidity and mortality in patients suffering from cancer [16]. Therefore, an effort was made to use the same route of drug administration to solve the drug bioavailability problem.

The buccal drug delivery system has numerous advantages. It is expected to overcome the problem of first-pass drug metabolism, which enhances the drug bioavailability, reduces the dose, and consequently reduces the side effect. Buccal films were useful in the treatment of chronic periodontitis [17]. In addition, the drug toxicity can be promptly terminated by removing the dosage from the buccal cavity. It is also possible to administer drugs to patients who cannot be dosed orally [18]. The high oral dose of the antimycotic drugs, which are used for oral candidiasis, could be reduced by using bioadhesive film to entrap the drug. Besides, the use of the penetration enhancer in combination with the antimycotic drug led to a dramatic reduction in its minimum inhibitory concentration (MIC) [19].

The preparation of CC as a bioadhesive film would be expected to enhance the drug bioavailability and reduce the fluctuation of drug concentration in the blood due to the following reasons: (i) First, bypassing the hepatic first-pass effect; (ii) Second, the adhesion of the film and its interaction with the buccal membrane will lead to a prolonged stay of the drug, increasing its release time in the blood. Accordingly, the aim of this study is the formulation and physicochemical evaluation of buccal bioadhesive CC films. The pharmacokinetic assessments of the drug from selected prepared bioadhesive films in rats, comparing with the normal drug oral dosage form used should be studied to prove the benefits of the suggested selected dosage form.

## 2. Materials and Methods

Acetonitrile HPLC grade, Methanol HPLC grade, and Orthophosphoric acid laboratory reagent grade were purchased from Fisher Scientific, UK. Candesartan cilexetil (CC) and carboxymethyl cellulose (CMC) were obtained as a gift sample from Sigma Pharmaceutical Company, Quesna, Egypt. Polyvinylpyrrolidone K40 (PVP K40) was purchased from Sigma Chemical Co., Steinheim, Germany. Propylene glycol (PG) was purchased from BDH chemical Ltd., Poole, UK. Tween 80 (TW) was purchased from El Nasr Pharmaceutical Chemicals Co., Cairo, Egypt. All other chemicals were of analytical grade and used as received.

### 2.1. Equipment

Electron scanning microscope [SEM] (JEOL-model: JSM-5200LV, Tokyo, Japan); FTIR (Tensor 27 Broker, Borken, Germany); Magnetic stirrer (VELP Scientifica, Usmate Velate, Italy, Europe); UV/visible spectrophotometer (Thermo Fisher Scientific, model EVO 300PC, software: vision pro, Carlsbad, CA USA); Paddle USP dissolution apparatus, Type Disc 6000 (Copley Scientific, Colwick, UK). HPLC (Thermo Scientific Dionex Ultimate 3000 UHPLC+ focused equipped with Dionex Ultimate 3000 fluorescence detector, Dionex Ultimate 3000 Column Compartment, Dionex Ultimate 3000 Pump, and Dionex Ultimate 3000 Autosampler. C18 chromatographic column (250 mm × 4.6 mm 5μ Thermo scientific BDS Hypersil) was used with a 1.5 mL/min flow rate), A combined glass electrode pH meter (Hanna instruments: microprocessor pH meter; pH 211; Smithfield, RI, USA), Digital balance OHAUS electric balance; model PA413; USA, Vernier Caliber (Poznań, Poland, 15 mm × 0.05 mm).

### 2.2. Preparation of CC Bioadhesive Buccal Film

The bioadhesive buccal films were prepared by the solvent casting method [20]. The prepared film compositions are reported in Table 1. The required amounts of CMC and PVP were dissolved in 30 mL of hot water (70 °C) to form a clear solution. The quantities of Tween 80 and propylene glycol were added while stirring. The required amount of the drug was dissolved in the prepared polymer solution. The solution was casted into a petri dish with a surface area of 63.642 cm^2^ and dried in the oven at 40 °C for 48 h. The prepared films were let to equilibrate with the room humidity at room temperature. The dry film was cut into square-shaped sections with an area that theoretically contains 16 mg of the drug.

### 2.3. Evaluation of the Prepared Buccal Film

#### 2.3.1. Instrumental Assessments of the Bioadhesive Films

##### Scanning Electron Microscopy (SEM)

The surface of the prepared selected films of F2, F5, and F7 was studied by using a scanning electron microscope. The selection is based on the films prepared by using the lowest, the middle, and the highest theoretical drug content. The magnification used depended on the best view to elucidate the presence or absence of drug crystal entrapment in the prepared film.

##### Fourier Transform Infrared Spectroscopy

The Fourier transform infrared (FTIR) of CC, the film plan, and medicated film prepared by using 40 or 240 mg drug were recorded using a FTIR spectrophotometer. Samples were mixed with potassium bromide (spectroscopic grade) and compressed into disks using a hydraulic press before scanning from 4000 to 600 cm^−1^.

##### Microenvironment pH

The palatability of the films was assured by measuring the microenvironment pH of the prepared buccal films. The films were soaked in 5 mL of distilled water for 1 h at ambient temperature. After equilibration for one minute, the pH of the surface was measured by mounting the electrode on the surface of the swollen film [19]. Triplicates of the experiment were performed.

#### 2.3.2. Physical Properties Assessments of the Bioadhesive Films

##### Weight Uniformity

The weight uniformity of the cut square-shaped sections of the same film was determined gravimetrically, according to Semalty et al. [21]. The weight of 6 samples from each film was determined using a digital balance. The results were analyzed for mean and standard deviation.

##### Thickness Uniformity

The square-shaped samples from each film were also used for the determination of the thickness of the prepared films via vernier caliber [22]. The results were analyzed for mean and standard deviation.

##### Folding Endurance

The folding endurance of the prepared films was determined according to that reported by Khairnar et al., [20]. This was done by repeating the folding of the prepared films at the same place. The end of the experiment was either the breaking point of the film or after it was folded 100 times without breaking.

#### 2.3.3. Drug Content Uniformity

A square-shaped area of each film that theoretically contains the same amount of drug was dissolved in 100 mL phosphate buffer pH 6.8 at 60 °C. One ml of the dissolved film solution was added to 4 mL of phosphate buffer pH 6.8. The resultant solution was measured spectrophotometrically at 256 nm. Triplicate experiments were performed. The actual drug content (ADC) was calculated and expressed as a percentage of the theoretical drug content (TDC) using the following equation:

Drug content (%) = (actual drug content/Theoretical drug content) × 100(1)

The results were analyzed for mean and standard deviation.

#### 2.3.4. Swelling Index

**Figure 1:**
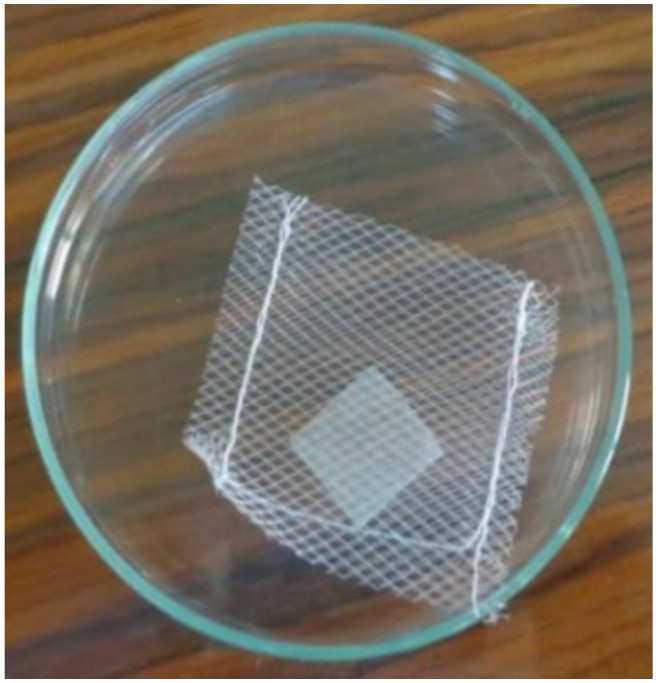
A special holder for the film sample.

A plastic thread mesh with a sieve opening of approximately 500 µm was used as a holder for studying the swelling index (Figure 1). The unloaded holder was immersed in phosphate-buffered at pH 6.8 for 5 min. The excess buffer solution was removed by gentle shaking and weighed. The film sample was placed in the holder and weighed at zero time. The loaded holder was immersed again in the buffer solution. After carefully removing any surface moisture, the loaded holder was reweighed at a preselected time interval. The swelling-erosion index was calculated using the formula [23]:

Swelling index = (Wt − W0)(2)

where Wt is the weight of the film at time t and W0 is the weight of the film at zero time.

#### 2.3.5. In Vitro Bioadhesion Strength

For measuring the bio adhesion strength, the rabbit intestine mucosal membrane was used as a model [24]. The research protocol and ethical guidelines were strictly followed according to the Institutional Animal Care and Use Committee (approval reference ZU-IACUC/3/F/81/2021). Male albino rabbits (n = 2, age about 10 weeks old, bodyweight 2.1–2.3 kg) were obtained from the animal house of the faculty of pharmacy, then were accommodated in a clean cage with free access to food and water. After overnight fasting, rabbits were euthanized (by IM injection of ketamine HCl), then the rabbit intestine was excised, washed gently with phosphate buffer pH 6.8, and cut longitudinally to expose the mucosal surface which was then again cut into rectangular pieces (4 cm^2^). These were glued with cyanoacrylate adhesive on the ground surface of a holder made of cellulose acetate plastic film so that the mucosal surface is uppermost. The buccal film was glued to another holder of the same size. The surface of the rabbit intestine was moistened with phosphate buffer pH 6.8. The rabbit intestine holder and buccal film holder were put in contact with each other with uniform and constant light pressure between fingers of the same person for one minute (preload time) to facilitate adhesion bonding [25]. The upper tissue holder was allowed to hang on an iron stand with the help of an aluminum wire fastened with a hook fixed on the back of the holder. A pre-weighed lightweight polypropylene bag was attached to the hook on the backside of the lower film holder with aluminum wire. After a pre-load time of one minute, water was added to the polypropylene bag using a burette adjusted to deliver water at a rate of 2.0 drops per second until the film was detached from the tissue. The collected water in the bag was weighed and expressed as the weight (gram) required for the detachment (bioadhesive strength) [25]. The force of adhesion and bond strength was calculated according to the following equations [26]:

Force of adhesion (N) = (Bioadhesive strength (g) × 9.81)/1000(3)

Bond strength (N m^−2^) = Force of adhesion/film surface area (4)

#### 2.3.6. In Vitro Bioadhesion Time

The time of the in vitro residence of different films was evaluated by assessing the time required for these films to detach from rabbit intestinal mucosa [27]. By using cyanoacrylate glue, the rabbit intestinal mucosa was fixed with mucosal side facing up on the surface of a glass slide coverslips. The mucosa was moistened with phosphate buffer solution (pH 6.6). The film (1 cm^2^) was wetted with the same buffer and pasted to the rabbit intestinal mucosa by applying a light force with a fingertip for one minute. The whole assembly was placed in the dissolution vessel so that the film is facing up and the glass side is down before adding 250 mL of phosphate buffer pH 6.8 previously equilibrated at 37 ± 0.5 °C. The dissolution paddle was rotated at a rate of 50 rpm. This stirring rate is believed to simulate the environment of the buccal cavity. The time taken for the film to completely erode or detach from the mucosa was recorded as the in vitro mucoadhesion time [28]. 

#### 2.3.7. In Vitro Release Study

The drug release from the films was conducted using USP rotating paddle dissolution test apparatus. The dissolution medium was 200 mL of phosphate buffer pH 6.8 with the controlled temperature at 37 ± 0.5 °C and a stirring rate of 100 rpm. A buccal film contains 16 mg of determining drug content was added to the dissolution media. Samples (5 mL) were withdrawn at predetermined time intervals and replaced with an equal volume of fresh dissolution medium. The samples were measured spectrophotometrically at 256 nm [29]. The same procedure was also carried out for our drug powder. In each case, three replicates were conducted. 

### 2.4. Bioavailability Study in Rats

#### 2.4.1. Rats 

A total of 24 male Wistar rats (240–260 g) were obtained from the animal house in the Faculty of Pharmacy, Zagazig University. The research protocol and ethical guidelines of the Zagazig University, School of Medicine’s Research Ethics Committee were strictly followed (approval reference ZU-IACUC/3/F/81/2021). All rats had unrestricted access to water and a normal rodent diet. Rats were randomly divided into four groups (6/group) to test the absorption of a single dose of 2.5 mg CC administered by four different pharmaceutical forms: (a) Oral group (OG) where an oral dose was ingested via gastric gavage after intraperitoneal (IP) anesthesia. (b) Film 40 group (F40) in which F40 film was inserted under the tongue of an anesthetized rat. (c) Film 160 group (F160) in which F160 was inserted at the buccal cavity of the rat, and (d) Film 240 group (F240) where F240 was used in the same manner. 

#### 2.4.2. Surgical Procedures

After overnight fasting, rats were injected with a mixture of ketamine (80 mg/kg) and xylazine (12 mg/kg) via the IP route [30]. After anesthesia, the skin of the dorsal and ventral aspects of the neck was shaved and sterilized with alcohol 70%. A surgical incision was made 3 mm to the right side of the midline on the ventral aspect of the neck, dissection of the subcutaneous tissues was done until reaching the groove between the trachea and sternomastoid muscle where gentile dissection was done to reach the carotid sheath. A special trocar was inserted via the incision to appear in the dorsal aspect of the neck, then a carotid catheter (catalog no CX-2012S, BASi Co, West Lafayette, IN, USA) was introduced into the trocar’s window to be withdrawn from the dorsal aspect down to the ventral one of the neck. A 3 cc syringe was used to fill the catheter lumen with heparinized saline. The rat was fixed in the supine position then the carotid artery was identified, separated from surrounding structures, and ligated by two ligatures; the distal one was tight while the proximal one was kept loose. A sterilized plastic strip (4 mm width) was inserted behind the carotid to support it. The bulldog clamp was applied proximal to the proximal loose ligature to stop bleeding until complete catheterization was finished. An iris scissor was used to produce a partial-cut of the wall of the carotid between the two ligatures then the tip of the carotid catheter was introduced inside the lumen of the carotid for a short distance then the loose ligature was tightened over the catheter inside the artery, then the Bulldog was removed and the catheter was further introduced inside the carotid. The plastic strip was removed, and the incision was closed in layers [31], while the dorsal hub of the catheter was fixed to the skin by two sutures. 

#### 2.4.3. Dosage and Blood Sampling 

The dorsal hub of the catheter was fixed to the harness which was fitted around the neck and forelimbs of the rat. The harness was connected to the Culex ABC tether and swivel system (BASi Co., West Lafayette, IN, USA). Designed films (F2, F5, and F7) or oral form with 2.5 mg dosage were inserted in the buccal cavity or via gastric gavage, respectively. The selected dosage of 10 mg/kg (2.5 mg/rat) was according to Dudhipala et al. [5]. Serial blood samples (200 μL/each) were withdrawn at 10 time-points (1, 2, 3, 4, 5, 7, 9, 11, 13, and 24 h) [32]. Blood samples were collected in heparinized mini collection tubes, centrifuged then the plasma was kept frozen until the time of analysis. 

#### 2.4.4. High-Performance Liquid Chromatography (HPLC) Assay 

Assay of candesartan as a base in serial plasma samples was carried out by applying of RP-HPLC analytical method developed by P.S.C staff in the Bioavailability Center of Faculty of Pharmacy, Tanta University. An amount of 2.3006 g of ammonium dihydrogen phosphate was dissolved in 1-L deionized water to prepare a 20 mM ammonium dihydrogen phosphate buffer. The pH value of the prepared solution was adjusted to be 3 by using orthophosphoric acid, then filtered and degassed. A mixture of ammonium dihydrogen phosphate buffer (pump A) and Acetonitrile (Pump C) with a ratio of 40:60 *v*/*v* was pumped as a mobile phase with a flow rate of 1.5 mL/min. 

The internal standard (Etodolac) preparation was done by dissolving 0.02 g in 100 mL methanol to prepare 200 μg/mL, then a sample was taken and diluted with methanol to prepare an internal standard stock solution of 10 μg/mL. 

For each 50 μL of unknown sample of the rat plasma, 5 μL of the internal standard were added, vortexed for 30 s, and then 100 μL of the acetonitrile added and vortexed again for 30 s. The prepared samples were centrifuged at 4000 rpm for 10 min. A 25 μL from the supernatant was injected on HPLC. The calibration range was 10 to 200 ng/mL.

#### 2.4.5. Pharmacokinetic Study 

MS Office Excel sheets 2003 were used to calculate the pharmacokinetic parameters in a one-compartment model. The peak plasma concentration of Candesartan (Cmax) and the time elapsed to reach it (Tmax) were determined by locating the concentration-time curves of different groups. Spreadsheets and their free add-on tools were used to calculate the concentration at zero time (C_0_), elimination rate constant (K_el_), absorption rate constant (K_ab_), and elimination half-life (t_1/2_). The area under the concentration curve from time zero to the time of the last measured time point was calculated by the linear trapezoidal method (AUC_0–t_ = (C_P0_ + C_P1_)/2 × (T_1_ − T_0_)) The AUC was concluded to infinity (AUC_0–__∞_) by the summation of AUC_0–t_ plus (C_last p_/*k*). Relative bioavailability of studied films was calculated as following (F_rel_ = AUC of a given film/AUC of oral × 100) [33,34]. 

### 2.5. Statistical Analysis

Noncategorical data were represented as mean ± SD. The pharmacokinetic parameters among study groups were compared using a Kruskal–Wallis H test (this nonparametric one-way ANOVA was used due to the relatively small sample size of the groups) with the LSD post hoc test for multiple comparisons, and *p*-values < 0.05 were considered statistically significant. The SPSS Statistical Package for the Social Sciences (SPSS, version 25; SPSS Inc., Chicago, IN, USA) was used for analysis. 

## 3. Results

### 3.1. Formulation 

The success of preparation of a bioadhesive film entrapped miconazole and penetration enhancer for the treatment of oral candidiasis [15] encouraged to use of the same placebo film to entrap candesartan with a significant structure-function change to be suitable for the new dosage form. PVP was selected to be used instead of using PVA to enhance the solubility of the drug [35], besides, to improve the mucoadhesion effect of the prepared film [25,36].

Table 1 represents the composition of the different CC buccal mucoadhesive films. A placebo film (F1) was also prepared for studying the effect of the presence of different concentrations of the drug on the physicochemical properties of the films. All films prepared using formulae from F1 to F6 were transparent, uniform, and flexible. When using 240 mg CC, the formed film was less transparent (F7). That may be due to using high drug concentrations. Increasing the concentration of tween 80 to between 150% and 200% led to the formation of a transparent film with an oily layer over the film (F8–F9). The film formation was inhibited by a change in the PVP-CMC ratio (F10–F11) and a jelly film structure was formed. 

**Figure 2:**
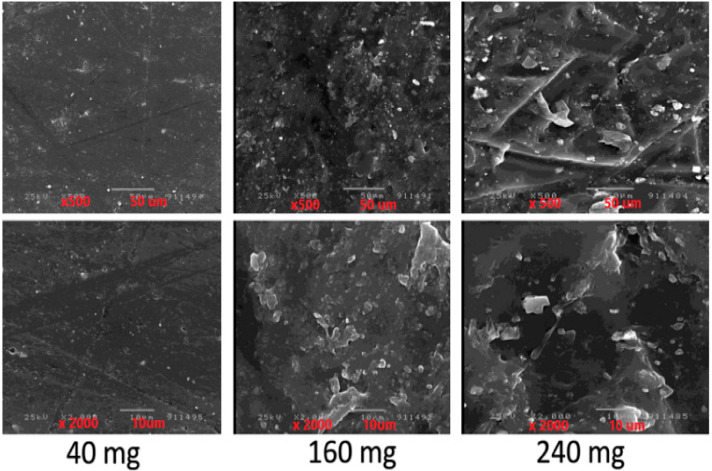
Electron scanning microscope image of selected prepared bioadhesive films.

Figure 2 showed the surface electron scanning of three selected bioadhesive films prepared by using different drug concentrations. From the image, a smooth film surface including the appearance of minute drug crystals deposited on the film prepared with a low drug concentration (40 mg) can be seen. This conclusion is also supported by the finding that increasing the image magnification (×2000) indicates the same shape. Increasing the drug concentration (240 mg) led to the loss of a smooth surface structure and the appearance of holes in the needle form. These needle holes may represent the hole places of the precipitated drug during preparation, which were lost during the processing of the dry film. Increasing the magnification of the same film (×2000) showed huge cavities for the lost drug crystals from the film surface. This may explain the lower transparency of the prepared film when using high drug concentration. Studying the image of the film prepared using 160 mg showed the same finding reported when using 240 mg with less effect. Consequently, it can be concluded that the drug entrapment method in the prepared bioadhesive film depends on the drug concentration used, which would be reflected in the drug release.

**Figure 3:**
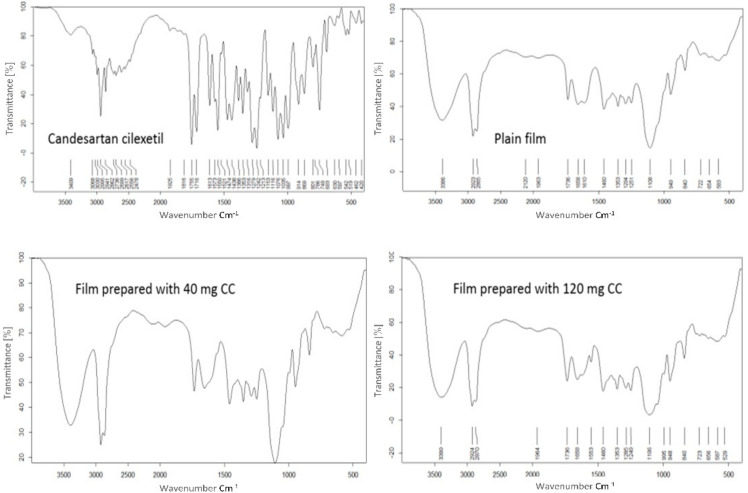
FTIR of CC, plain film, and different selected prepared films for comparison.

The placebo film components and also those containing different concentrations of the pure drug were clear solutions during preparation. Therefore, chemical interaction between the drug and the other film components could be possible. The formed films were clear and transparent. Accordingly, FTIR scans of the pure drug, placebo film, and selected different films prepared by using different drug concentrations were carried out (Figure 3). The characteristic peaks of the ideal pure drug powder could be seen at 2941 cm^−1^ for aromatic (−C−H) stretching, 2862 cm^−1^ for (O−H) stretching, 1755 cm^−1^ and 1716 cm^−1^ forester (−C=O) stretching vibration, 1279 cm^−1^ and 1316 cm^−1^ for (−C−O) stretching of the carbonyl group of aromatic esters and 749 cm^−1^ for the substitution aromatic ring [37]. At the time, it is difficult to interpret the FTIR scan of the placebo polymer film, which may be due to the multicomponent of the placebo film. For example, the prominent peaks of PVP could not be assigned [37]. Comparing the FTIR scan of the ideal pure drug and that of the placebo polymer film can distinguish most of the characteristic peaks of the ideal pure drug. That is due to the components of the placebo film that have no peaks where the peaks of the ideal pure drug exist. On contrary, all characteristic peaks of the ideal pure drug completely disappeared in the FTIR scan of the films containing different concentrations of the drug (Figure 3). That may be due to changing the total symmetry of the drug molecules as a result of its molecules’ entrapment in the polymer molecules [15]. Besides, it was reported that the trapping of the drug molecules inside the oily core matrix led to the absence of the drug minor peaks [38]. At the same time, the drug major characteristic peaks at 1736 cm^−1^ and 1249 cm^−1^ which represent the ester (−C=O) carbonyl stretching of the drug and (−C−O) stretching in aromatic ester respectively, could be assigned. The presence of the drug’s major characteristic peaks may be indicative of the absence of interaction between the drug and the polymer [36,39]. The shifting of the ester (−C=O) stretching vibration from 1713 cm^−1^ to 1736 cm^−1^ may be due to the conversion of the drug crystal form to an amorphous form [36,40]. In general, it could not be concluded that these drug characteristic peaks in the prepared films are due to the drug only because they may also be seen in the placebo film.

The physicochemical characteristics of the prepared films were studied, and the results are summarized in Table 2. It should also be reported that the results were calculated at per one cm^2^. That is to facilitate the comparison and studying the effect of the different drug concentrations on the physicochemical characteristics of the prepared films. The weight uniformity of one cm^2^ from different films was found to be increased by increasing the theoretical drug content. At the same time, the actual drug content in the one cm^2^ of the different films was also increased by increasing the theoretical drug content up to 160 mg per film and then decreased on using 200 mg (F6) and 240 mg (F7) per film. That may be due to the surface precipitation of the drug crystal on the film, which could be lost during film processing and seen by film surface electron scanning. The values of standard deviation showed a small variation in weight and actual drug content which indicates the efficiency of the method used. These results were also reflected in the thickness of the prepared films which is increased by increasing the theoretical drug content. The film thickness ranged from 0.30 to 0.52 mm indicating the ideality of the prepared mucoadhesive buccal films.

The folding endurance was found to be very high indicating the flexibility of the films which was observed from the capability of the films to tolerate the folding several times without cracking. Besides, the flexibility of the prepared mucoadhesive buccal films is essential to be easily applied on the site of application. 

Since the prepared film is a mucoadhesive buccal film, it was essential to simulate their palatability by measuring the pH of the microenvironment of different batches. The adhesion of the film and its solubility may change the buccal pH. Besides, changing the mouth pH and remaining sometimes in the mouth, a negative effect on the mouth flora could be expected. Measuring the film surface pH, it was found that, pH value increased by increasing the theoretical drug content and the pH ranged from 6.68 to 7.23 indicating the palatability of the prepared films and there is no awareness from the damage of the oral mucus membrane [15,19].

**Figure 4:**
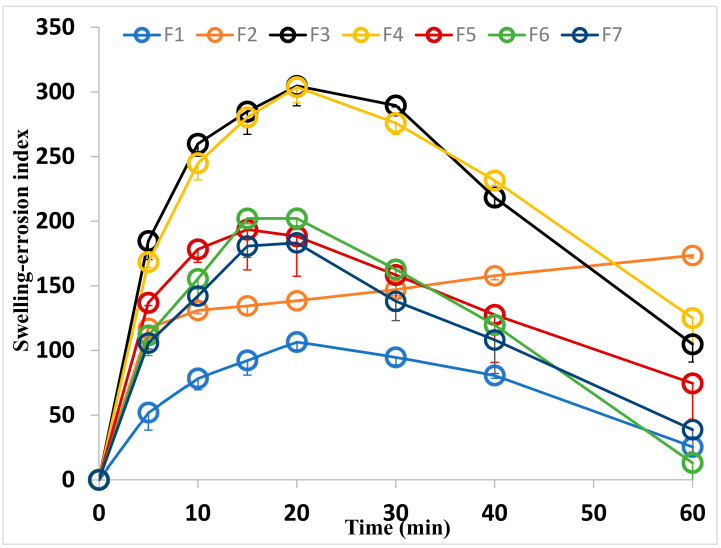
Swelling index of the placebo and medicated bioadhesive films papered by using different drug concentrations.

Water absorption capacity or swelling capacity is an important factor for the bio-adhesion property of the film to facilitate the drug release which is mainly done by diffusion and erosion. Therefore, the water absorption capacity and erosion of the polymer are two evaluating tests that should be carried out for the prepared films. Water diffusion into the matrix leads to its hydration, swelling and then the drug diffuses out. Besides, the polymer erosion could be expected to occur simultaneously. At predetermined time intervals, the weight of the film should be determined. In the case of increasing the weight of the film as a result of its hydration, this phase represents the swelling phase although both diffusion and erosion have occurred simultaneously with predominant swelling. The opposite should be expected in the case of decreasing the weight of the film. The same procedure was applied to the buccal bioadhesive CC films (Figure 4). From the figure, it can be noticed the swelling, diffusion, and film erosion phases. The swelling phase of the films prepared on using the drug is markedly higher than that of the placebo film and this phase occurred during the first 20 min. The swelling phase increased with increasing the drug concentration used and then decreased. The same results could be also noticed in the erosion phase. For the medicated film prepared using a 40 mg drug, there is a swelling phase. Since the difference between the prepared films is the presence or absence of the drug and its concentration, accordingly it can be concluded that that is due to the drug entrapment in the polymer matrix. Since the drug has a lower solubility in the dissolution media, then, it can be suggested that the presence of the drug molecules leads to relaxing the matrix polymer chain. These created channels giving the chance for hydration and diffusion of the matrix and consequently swelling. Increasing the drug concentration may lead to decreases in the channels which resulted in decreasing the swelling of the film. Besides, the lipophilicity of the drug could also lead to decreasing in the swelling of the film.

Table 3 represents the results of studying the mucoadhesion of the prepared films to the buccal mucosa. From the table, it can be seen that the bioadhesion strength and consequently adhesion force and bond strength increased by increasing the amount of drug used in the film preparation.

**Figure 5:**
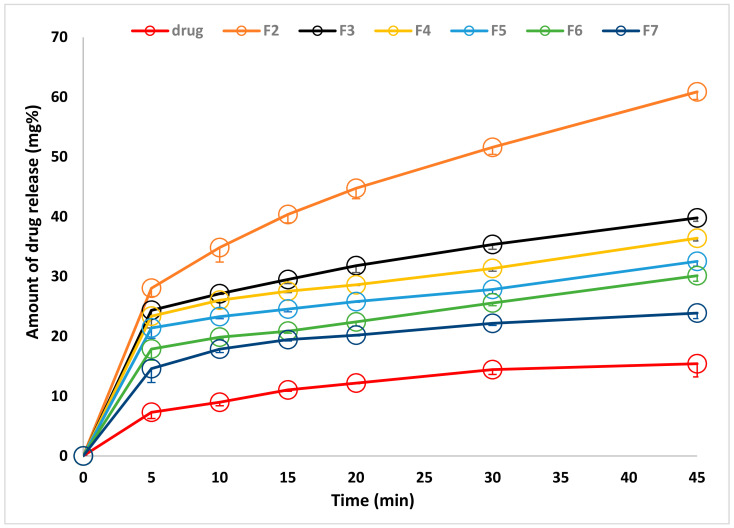
Drug release profile from different mucoadhesive films prepared by using different drug concentrations and pure drug.

The drug release profile from the prepared dosage form represents the last step invitro stage. It stimulates the release of the drug after administration. Therefore, it should be carried under standard procedures suggested by the pharmacopeia. Figure 5 shows the drug release profile of the pure drug in powder form and from different medicated films containing the same concentration of the actual drug content. From the figure, it can be seen that the drug release profile from pure drug powder is lower than that from all prepared bioadhesive buccal films. In addition, all drug release profiles have a burst effect and incomplete drug release. The rapid initial drug release increased by decreasing the actual drug content. At the same time, the amount of incomplete drug release increased by increasing the actual drug content. The drug release profile from different films and pure drug powder could be arranged as the following: from film prepared on using 40 mg > 80 mg > 120 mg > 160 mg > 200 mg > 240 mg > pure drug. 

### 3.2. Bioavailability Study

**Figure 6:**
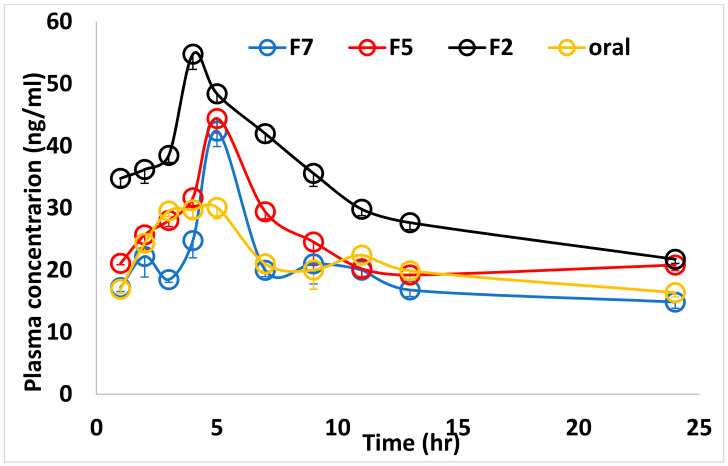
Means of plasma concentrations of Candesartan at different time points among groups.

The plasma concentration-time curve for different forms of CC after a single dose of 2.5 mg was shown in Figure 6, and the mean pharmacokinetic parameters were summarized in Table 4. The AUC 0–∞ was relatively higher in F160 and F40 than in oral form (*p* ≤ 0.001). Compared to the oral form, the Cmax was significantly higher in the F40, F160, then F240 (*p* ≤ 0.001). The high Cmax of F40 was associated with significantly lower t_1/2_ and higher absorption rate constant.

## 4. Discussion

The CC is an antihypertensive drug with absolute bioavailability of about 14–40% [1]. The low and wide range of the drug’s absolute bioavailability may be due to the drug having three different biopharmaceutic properties. From the biopharmaceutic view, the drug is a class II which indicates that the drug has low solubility. Drug solubility is the first step in the drug absorption process. The drug absorption from GIT has also an absorption problem since the drug is P-gp substrate (P-glycoprotein) with high P-gp efflux [41,42]. Besides, CC undergoes extensive first-pass metabolism in the liver when administered as an oral convenient dosage form [5]. 

To avoid the hepatic effect on CC (first pass mechanism), the idea was directed to use a bioadhesive buccal film dosage form, which could solve the drug administration problem. Besides this, it is also known that the expression of P-gp in mouth mucosa is low [17]. The drug solubility could be increased by its molecular dispersion in a hydrophilic polymer. Therefore, the formulation of CC in a bioadhesive buccal film would be expected to improve its bioavailability. This hypothesis could be reported since the bioadhesive buccal films especially the F2 showed better AUC. The maximal concentration of buccal films especially F2 were significantly higher than the oral equivalent dose. Furthermore, the F2 film showed a significantly higher absorption rate (0.66 ± 0.02 for F2 vs. 0.30 ± 0.01 for the oral, *p* < 0.001)) even more than the other formulated films (F5 and F7). This could be explained by the fact that in F2 film the medication was entrapped in the molecular and/or minute drug crystals which result in easy disintegration, solubilization, and diffusion. This results in the highest initial drug release. Interestingly, this drug burst was followed with significantly lower K_el_ and t_1/2_ values when compared with oral form or other buccal films. The t_1/2_ in studied forms was like that was reported by Zhang et al. [43] (≈2 h). However, different values were published such as 9 h [41], or a longer half-life of 29 h. in hypertensive patients [42], or even shorter (≈7 h) [5]. Using different species of medication with different degrees of purity or different formulation may give an interpretation of these discrepancies. 

In most cases, the preparation of a transparent film may indicate the molecular entrapment of the drug in the polymer. This leads to increasing the drug solubility in the case of using a hydrophilic polymer. Increasing the drug concentration leads to saturation of the polymer with the drug molecules. If the concentration of the drug further increased, another drug entrapment mechanism started as minute drug crystals. The amount of the drug entrapped in the molecular states depends on the physicochemical characters of both drug and film basic polymers. Entrapment of the drug crystals forms led to decreasing in the transparency of the film and decreasing the drug solubility and consequently bioavailability than that in the molecular state [15]. This effect could be noticed by decrease the transparency of the prepared film as in the case of using 240 mg of the drug and could be also accurately studied by using SEM for the prepared films. During film processing, the surface drug crystals could be loosed and appear as a hole in the body of the dried form as reported by studying the SEM images. To improve the drug entrapment method and decrease its crystallinity, two well-known trials were carried out. First, increasing the surfactant concentration led to the appearance of an oily layer over the dosage form. This created a production problem; besides, this layer may entrap the excess drug which opposes the idea of its application. The second is by changing the basic film polymer by increasing the concentration of PVP. This modification of the basic film polymer led to losing the dosage form architecture. That is due to the nature of PVP as a hygroscopic substance. 

The drug entrapment mechanisms were also reflected on the thickness and drug content of the prepared film due to its entrapment in the polymer architecture in minute drug crystals or as drug crystals attached to the surface [21,22]. In each case, this led to increasing the thickness of the prepared film comparing to the placebo. Increasing the theoretical drug content led to parallel increasing the actual drug content if the drug was entrapped in the minute drug crystal state (15). Saturation of the polymer with the drug minute drug crystal led to the next entrapment mechanism which is drug crystals. The drug crystals attached to the surface which, maybe, loosed during the film preparation process. Therefore, the actual drug content would be decreased with the appearance of deep holes, cave surface film structure and that is what is noticed in the case of bioadhesive films prepared by using 200 mg and 240 mg theoretical drug content.

The palatability of the prepared films may be also due to the drug entrapment mechanisms. The molecular state entrapment of a drug or its minute drug crystals in the polymer architecture lets to covering the drug molecular away from the surface. In other words, does not affect the surface pH. The film surface drug crystal, which formed because of increasing the theoretical drug content, has also no effect on the surface pH because of the drug’s low solubility [1,20]. 

The goal of the selected dosage form is to increase the drug residence time in the buccal mucus membrane to avoid the first pass mechanism by its nearly all absorption through the buccal way. This is thought to achieve by using a bioadhesive film as a dosage form. The bioadhesive force of the mucoadhesion films depends on molecular weight, swelling behavior of the polymers, and contact time with mucus. The bioadhesion characteristics of the prepared films are affected by the types and the ratios of the bioadhesive polymers used in the film preparation [44]. CMC was selected as one of the film’s basic bioadhesive polymers. It is an anionic polymer that gives the highest bio-adhesive force. The addition of PVP to the mucoadhesive film increases its bioadhesive strength due to the ability of PVP to form hydrogen bonding and Van der Waals forces with the mucous membrane [28,45]. Besides, the presence of PVP in the prepared mucoadhesive film enhances its swellability because of its water solubility and hygroscopic character [38]. On the other side, the components of the prepared film are constant, and the drug used is not water-soluble, which is not in agreement with increasing the bioadhesion force with increasing drug content. Surface electron scanning of the prepared films showed the formation of an empty needle places on the film surface indicating the loss of drug crystals during processes leaving the unsmooth waved surface. This led to increasing the film surface area and consequently its bioadhesive force [46,47].

The drug release profile is a simulating process to what would happen after using the dosage form. The drug release profile of the pure drug was the lowest one although it is done from pure drug powder form which is supposed to have a high surface area. This statement is based on the fact that increasing drug surface area by decreasing its particle size led to increasing the drug dissolution rate. Since the drug release profiles from all prepared films are higher than that of pure drugs, it can be stated that encapsulation of the drug in all prepared films led to decreasing the drug particle size than that of the pure drug, which is reflected on the drug release profile. This directed again to the relation between the drug entrapment mechanisms and the drug release profile. On using 40 mg drug concentration, the drug is entrapped in the molecular and minute drug crystals which easiest the drug molecules to dissolve and diffuse to the dissolution media. This led to higher rapid initial drug release (burst effect) and a lower amount of incomplete drug release since it was proved that there is no chemical interaction between the drug molecules and the basic film polymers [15,37]. Increasing the drug concentration in the preparation process leading to saturate the polymer with the drug molecules and another encapsulation mechanism started to be formed which is minute drug crystals. The size of the drug crystal would increase by increasing the drug concentration used which is proved by using ESM. The presence of the drug crystal, which needs to be dissolved first before diffusion to the dissolution media, led to a decrease in the rapid initial and increase in the amount of incomplete drug release. Besides the lower size of the drug crystals entrapped in the prepared films than that of pure drug, which led to a higher drug release profile, the encapsulation of the drug crystal in the hydrophilic polymer increases the drug crystal wettability and then solubility [40].

Mucoadhesive buccal films of CC are comparable with other recent formulations that targeted enhancement of CC bioavailability. Anwar et al. [48] created a loaded nanostructured-lipid carrier for CC with bioavailability double that of the oral suspension. The use of P-gp inhibitors is another technique [49]. Natural P-gp inhibitors (e.g., piperin and quercetin) enhanced CC bioavailability in rats by 68% and 27 and when quercetin and piperin, respectively, were used [50]. Another CC-loaded self-nano-emulsifying drug delivery system was developed to enhance CC bioavailability via the inhibition of intestinal P-gp transporters. However, they found that P-gp-mediated efflux having a minor effect in the oral bioavailability of CC [51]. Liquid-fill hard gelatin capsule technique was also used for improving the bioavailability of CC with a promising percentage of drug release [52]. Being of good bioavailability, easy application, and good tolerance by patients, it might be expected that the future CC formulation will be in form of mucoadhesive buccal films.

## 5. Conclusions

This study represents a prove to the suggested hypothesis, which is the use of a bioadhesive buccal film as a dosage form to solve some problems facing oral drug administration. The selection of the suitable polymers-based film and drug concentration led to the preparation of a buccal film dosage form. Besides, it can also increase the buccal drug residence time to achieved higher bioavailability of the drug. That means bioadhesion buccal films as a dosage form has a better bioavailability improving processes for a drug like CC: by avoiding the first hepatic bypass mechanism and increasing the drug absorption through the buccal way. This was clear in pharmacokinetic parameters especially the high Cmax and AUC for bioadhesion buccal films (F2) when compared with oral form. The ease of the preparation process, quality control, robustness, and ruggedness of the preparation process suggest the same procedure for drugs facing the absorption problems.

## Figures and Tables

**Table 1:** Composition of different CC buccal muco-adhesive films.

Formulae	CC (mg/Film)	CMC * (mg/cm^2^)	PVP * (mg/cm^2^)	Tween (mg/cm^2^)	PG * (mg/cm^2^)
F1	0	7.50	2.50	1.30	2
F2	40	7.50	2.50	1.30	2
F3	80	7.50	2.50	1.30	2
F4	120	7.50	2.50	1.30	2
F5	160	7.50	2.50	1.30	2
F6	200	7.50	2.50	1.30	2
F7	240	7.50	2.50	1.30	2
F8	240	7.50	2.50	1.95	2
F9	240	7.50	2.50	2.60	2
F10	240	5.00	5.00	1.30	2
F11	240	2.50	7.50	1.30	2

* CMC: carboxymethylcellulose; PVP: polyvinylpyrrolidone; PG: propylene glycol.

**Table 2:** Physical properties of the prepared CC buccal mucoadhesive films.

Formulae	Weight/cm^2^	* ADC/cm^2^	* F. Endurance	Thickness	pH
F1	28.33 (±2.36)	---------------	100	0.30 (±0.00)	6.680
F2	30.49 (±1.75)	0.58 (±0.017)	100	0.47 (±0.02)	6.740
F3	35.32 (±2.05)	1.04 (±0.029)	100	0.49 (±0.01)	6.760
F4	39.75 (±2.56)	1.43 (±0.080)	100	0.50 (±0.00)	6.790
F5	41.48 (±1.93)	1.92 (±0.075)	100	0.50 (±0.02)	6.900
F6	41.90 (±2.50)	1.73 (±0.045)	100	0.52 (±0.02)	7.160
F7	42.34 (±2.45)	1.63 (±0.075)	100	0.52 (±0.03)	7.230

* ADC: Actual drug content; F. endurance: Folding endurance.

**Table 3:** Bioadhesion strength, adhesion force, and bioadhesion time of the different prepared bio-adhesive films.

	Bioadhesion Strength (g)	Adhesion Force (N)	Bond Strength (Nm^2^)	Bioadhesion Time (min)
F1	31.144 (2.40)	0.306(0.01)	0.076	55(2.10)
F2	32.437 (1.80)	0.318(0.03)	0.080	58(3.40)
F3	32.504 (2.02)	0.319(0.03)	0.080	59(2.20)
F4	36.147 (3.12)	0.355(0.02)	0.089	61(2.90)
F5	36.934 (1.15)	0.363(0.04)	0.092	64(3.60)
F6	39.932 (4.20)	0.392(0.02)	0.098	66(1.50)
F7	42.396 (3.12)	0.416(0.03)	0.104	70(1.90)

**Table 4:** Pharmacokinetic parameters among study groups.

Parameters	Oral	F2 (40 mg)	F5 (160 mg)	F7 (240 mg)	Sig
AUC_0–∞_ (ng * h/mL)	547.39 ± 7.30 ^a^	972.89 ± 7.29 ^b^	544.16 ± 7.62 ^a^	509.98 ± 6.53 ^c^	<0.001
C_max_ (ng/mL)	30.03 ± 1.57 ^a^	54.47 ± 3.13 ^b^	44.40 ± 2.55 ^c^	42.37 ± 1.66 ^c^	<0.001
T_max_ (h)	5.00 ± 0.24 ^a^	4.00 ± 0.16 ^b^	5.00 ± 0.30 ^a^	5.00 ± 0.29 ^a^	<0.001
K_el_ (h^−1^)	0.29 ± 0.02 ^a^	0.10 ± 0.02 ^b^	0.29 ± 0.03 ^a^	0.29 ± 0.01 ^a^	<0.001
K_ab_ (h^−1^)	0.30 ± 0.01 ^a^	0.66 ± 0.02 ^b^	0.30 ± 0.02 ^a^	0.31 ± 0.02 ^a^	<0.001
t_1/2_ (h)	2.22 ± 0.16 ^a^	1.05 ± 0.03 ^b^	2.17 ± 0.97 ^a^	2.18 ± 0.22 ^a^	<0.001
F_rel_	Reference	177.73%	99.41%	93.17%	-

Sig is the significance; Different superscripts (a, b, and c) mean statistically different. AUC_0–__∞_ is the area under the concentration curve from time zero to infinity; Cmax is the peak plasma concentration of CC; Tmax is the time elapse to reach Cmax; K_el_ is elimination rate constant; K_ab_ is the absorption rate constant; t_1/2_ is elimination half-life; F_rel_ is the relative bioavailability (AUC of a given film/AUC of oral).

## Data Availability

All data are available upon reasonable request from the corresponding authors.
